# Probability‐Distribution‐Configurable True Random Number Generators Based on Spin‐Orbit Torque Magnetic Tunnel Junctions

**DOI:** 10.1002/advs.202402182

**Published:** 2024-04-15

**Authors:** Ran Zhang, Xiaohan Li, Mingkun Zhao, Caihua Wan, Xuming Luo, Shiqiang Liu, Yu Zhang, Yizhan Wang, Guoqiang Yu, Xiufeng Han

**Affiliations:** ^1^ Beijing National Laboratory for Condensed Matter Physics Institute of Physics University of Chinese Academy of Sciences Chinese Academy of Sciences Beijing 100190 China; ^2^ Songshan Lake Materials Laboratory Dongguan Guangdong 523808 China

**Keywords:** bayesian network, magnetic tunnel junctions, spin‐orbit torque, true random number generator

## Abstract

The incorporation of randomness into stochastic computing can provide ample opportunities for applications such as simulated annealing, non‐polynomial hard problem solving, and Bayesian neuron networks. In these cases, a considerable number of random numbers with an accurate and configurable probability distribution function (PDF) are indispensable. Preferably, these random numbers are provided at the hardware level to improve speed, efficiency, and parallelism. In this paper, how spin‐orbit torque magnetic tunnel junctions (SOT‐MTJs) with high barriers are suitable candidates for the desired true random number generators is demonstrated. Not only do these SOT‐MTJs perform excellently in speed and endurance, but their randomness can also be conveniently and precisely controlled by a writing voltage, which makes them a well‐performed Bernoulli bit. By utilizing these SOT‐MTJ‐based Bernoulli bits, any PDF, including Gaussian, uniform, exponential, Chi‐square, and even arbitrarily defined distributions can be realized. These PDF‐configurable true random number generators can then promise to advance the development of stochastic computing and broaden the applications of the SOT‐MTJs.

## Graphical abstract

Randomness is crucial for solving complex problems, neuron networks, and simulated annealing. Spin‐orbit‐torque magnetic tunnel junctions (SOT‐MTJs) are high‐performance Bernoulli bits precisely controlled by writing voltages. They are further used to sample truly random numbers with any probability distribution function such as Gaussian, uniform and exponential. These generators can support stochastic computing and expand the uses of SOT‐MTJs.

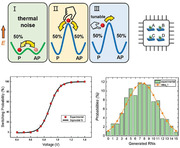

## 1 Introduction

Classic computing systems have been elaborately designed from reliable materials and devices to error‐correcting circuits and architectures to fault‐tolerant systems and algorithms in the fight against randomness and unpredictable behaviors when they are used in mundane automating tasks and solving complex scientific problems.^[^ ^1^ ^]^ Nevertheless, it is growingly appreciated to embrace randomness instead of enforcing determinacy for naturally stochastics‐preferred tasks such as Monte Carlo sampling,^[^ ^2^ ^]^ Bayesian neural networks (BNNs)^[^ ^3^ ^]^ and simulated/quantum annealing algorithm accelerators,^[^ ^4^ ^]^ not to mention the generative artificial intelligence^[^ ^5^ ^]^ and even artificial brain simulations^[^ ^6^ ^]^ in the coming future. Though stochastics with required probability distribution functions (PDF) can be introduced at the software levels in precision‐supreme computers,^[^ ^7^ ^]^ this strategy is undoubtedly burdensome in power efficiency, speed and parallelism, and bottlenecked by the memory wall issue in the current computing architecture. If applications are further demanding in the throughput, forms and distributions of randomness, pseudo‐random numbers generated by some fixed algorithms^[^ ^8^ ^]^ can even be unacceptable. In this scenario, true random number generators (TRNG) with tunable PDF integrated at the hardware level can be an option.

TRNGs have already played a key role in the applications of cryptography and data security^[^ ^9^ ^]^ and will continuously thrive with the increasing appeal for privacy protection for the sake of their non‐clonability. For these usages, TRNGs can be developed in many technique routes such as photonics and quantum fluctuations;^[^ ^10^ ^]^ however, for stochastic computing, types of randomness, PDFs, quality and quantity of generated random numbers all matter. For example, 10^15^ random numbers per second are needed to activate human–brain inspirations in conventional computing systems,^[^ ^1^, ^11^ ^]^ which has already raised rigorous restrictions for the size (or capacity), endurance, speed, and power of the desired TRNGs. Moreover, for such typical applications as the BNNs which have achieved great success in generative artificial intelligence recently,^[^ ^5b^ ^]^ types and PDFs of random numbers are influential. Weights in a BNN are stored in the form of random variables with tunable PDFs rather than scalars. Thus, even a BNN with only several weights can represent billions of networks in probability, which accounts for its extraordinarily strong ability to express reality. In practice, weights in a BNN are usually stored by random variables with the Gaussian PDFs for computational simplicity.^[^ ^12^ ^]^ Once generated according to the optimized PDFs, preferably, the random weights are nonvolatilely stored on‐site to facilitate their subsequent invocations. These random weights with configurable PDFs set a stringent requirement for hardware TRNGs to implement them.

A magnetic tunnel junction (MTJ) whose magnetic configuration and resultant resistance becomes thermally stochastic as preactivated to a critical state can be a promising candidate for an ideal TRNG.^[^ ^13^ ^]^ Here we experimentally demonstrate spin‐orbit torque (SOT) MTJs with 50 × 200 nm sizes, 300 ps writing speed, above 100% tunneling magnetoresistance (TMR) ratios at room temperature and over 10^12^ write endurance. The switching probability of a SOT‐MTJ is continuously controlled by the bias voltage, qualifying it as a tunable Bernoulli random number generator, a [0, 1] binary bit with tunable probability *p* (1‐*p*) to sample 1 (0). Furthermore, inspired by the Bayesian network method, we utilize this *p*‐tunable feature to realize TRNGs with configurable PDFs (Uniform, Gaussian, Exponentials, Chi‐square, and even arbitrarily defined distributions), which can well match the needs of the stochastic computing applications and also broaden the application scopes of the SOT‐MTJ devices.

## 2 Results and Discussion

**Figure 1:**
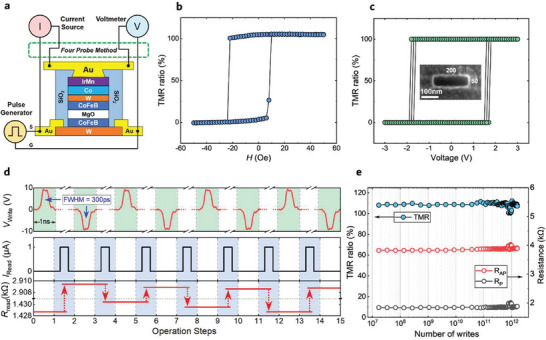
Characterization of the performance of current‐driven field‐free switching of the SOT‐MTJ. a) Device structure and measurement schematics. b) *R*‐*H* loop obtained by scanning the in‐plane magnetic field along the easy axis of the MTJs. c) Field‐free magnetization switching driven by 6 ns pulse currents. Inset is the top view of the scanning electron microscope (SEM) image of an MTJ. d) Field‐free magnetization switching driven by 300 ps pulses. e) over 10^12^ write endurance tests at 2.4 times the critical write voltage.

**Figure 2:**
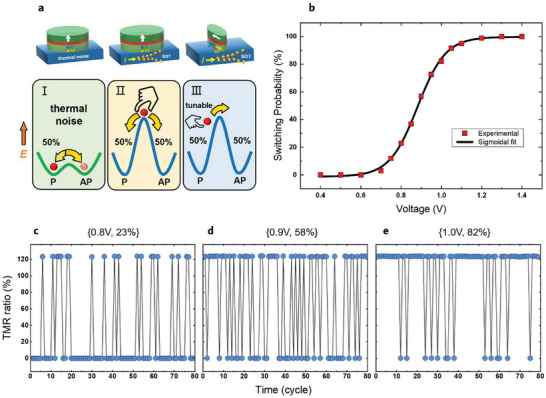
Experiment demonstration of MTJ‐based tunable TRNGs. a, Several different implementations of the MTJ‐based TRNG. b, MTJ switching probability versus write voltage. The black solid line is the fitted sigmoidal curve. c–e) the resistance results obtained from continuous testing at a certain voltage of c) 0.8, d) 0.9, and e) 1.0 V.

As shown in **Figure** **1**a, the MTJ device consists of the W/CoFeB/MgO/CoFeB‐synthetic antiferromagnetic pinned multi‐layer structure and the Au electrodes. Its resistance state is read out by the four‐terminal method with Keithley 2400 and 2182 sourcing a current and detecting the voltage across the MTJ, respectively. The MTJ can be switched between the parallel (low resistance) and antiparallel (high resistance) state by an external magnetic field (3D magnetic field probe station, East Changing Technologies, China) or a pulse current (Figure 1b,c). Specifically, the *R*‐*H* loop measured along the easy axis of the MTJ shows its high TMR ratio reaching above 100% (Figure 1b). The inset of Figure 1c shows that the MTJs have been well‐defined as 50 × 200 nm rectangles. The field‐free switching of the MTJ driven by a 6 ns pulse writing current was achieved (Figure 1c). The current‐induced switching degree is 100%, compared with the field‐induced switching. Worth noting, 1) pulses as short as 300 ps can also effectively drive the switching of the MTJ on the field‐free condition (Figure 1d); 2) Figure 1e experimentally demonstrates that our MTJ device has an endurance of over 1.6 × 10^12^ at a write voltage of 2.4 times the critical switching voltage. These performances of the MTJs such as the high TMR ratios above 100%, the small unit size and especially the ultrahigh writing speed and endurance, companied by their compatibility with the sophisticated magnetic random‐access memory and CMOS technologies, are very beneficial for them to be applied as the TRNGs with large throughput.

MTJ‐based TRNGs whose randomness all results from thermal fluctuations can be categorized into three types according to their service conditions (**Figure** **2**a). As MTJs have a *k*_B_ *T*‐comparable barrier, their resistance can automatically hop between a high and a low resistance state only under thermal activation without external stimulus. These low‐barrier MTJs thus have a low driving power but cannot remember generated random numbers. This kind of MTJs has been utilized as probabilistic pits (*p*‐bit) in integer‐factoring hardware.^[^ ^13^, ^14^ ^]^ For applications such as the BNNs, TRNGs must store generated weights for subsequent invocations, which calls for high‐barrier MTJs with nonvolatility. In this case, an MTJ can only produce random numbers when it is initially preset at its critical switching condition by spin‐transfer torques (STT) or spin‐orbit torques (SOT). The latter is advantageous in endurance and speed as demonstrated in Figure 1. Especially, the critical condition and as‐induced switching possibility can thus be tuned by the imposed SOT. As shown in Figure 2a, there are at least two ways to develop the SOT‐MTJ‐TRNGs with perpendicular magnetic anisotropy or in‐plane magnetic anisotropy. For the former, a large enough SOT first pulls the magnetization of the free layer into the plane (or the summit in the energy landscape). After releasing the torque, the perpendicular layer naturally relaxes to its spin‐up or spin‐down states with equal probability.^[^ ^13i^ ^]^ For the latter, by biasing a writing voltage close to the threshold *V*_c_, we can first activate the MTJ to a certain critical state where the significance of thermal fluctuation or the switching probability can thus be continuously tuned. For this reason, we are motivated to explore the configurable TRNGs based on the latter SOT‐MTJs with the in‐plane anisotropy.

When measuring the switching probability, we first used a current pulse to reset the MTJ to its low‐resistance state. Then an applied voltage was attempted to switch the magnetization of an MTJ to its high‐resistance state. The probability of switching the MTJ from the low‐resistance state to the high‐resistance state, namely, the switching probability, is dependent on the applied voltage. Choosing a certain voltage and repeating the above preset and write operations at this voltage 100 times will stochastically give out the switching probability of this specific voltage. The relation between the switching probability and the applied voltage is then obtained and shown in Figure 2b, which can be well‐fitted by a sigmoidal function. Figure 2c–e exhibits the output resistance of an MTJ working at 0.8, 0.9, and 1.0 V corresponding to the switching probability of 23%, 58%, and 82%, respectively, manifesting the accurate tunability of stochasticity of the SOT‐MTJ‐based TRNG. The random numbers generated at the 0.5 switching probability, after a simple and common XOR post‐processing, can pass all the 15 NIST randomness test toolsets (Figure S1, Supporting Information), displaying the high quality of this kind of TRNGs.

Till now, we have already demonstrated the SOT‐MTJs can be used as a Bernoulli bit, a binary TRNG with its switching probability (*p*) controlled by the bias voltage. However, instead of binary bits, (quasi‐) continuous TRNGs with configurable PDFs are expected for wider practical applications such as the BNNs whose weights are usually not binary. Furthermore, these random numbers complying to a required PDF generated at the software level are usually transformed from a uniform PDF between 0 and 1, any real number ∈ [0,1] being possible. However, here, the random numbers generated by the high‐barrier SOT‐MTJs belong to the Bernoulli style, which calls for a different protocol for the transformation from the algorithm currently applied in the software. Besides, if the process of transforming arbitrary PDFs is executed at a software level, it becomes inevitable to move hardware‐generated random numbers into computing units and process them there according to a prefixed algorithm such as the Box–Muller method for the Gaussian PDF from a uniform one^[^ ^15^ ^]^ and then deliver the transformed random numbers back to somewhere needed for the coming usages. Converting uniform random numbers into PDF‐specific random numbers by algorithms such as the cumulative distribution function (CDF) algorithm inevitably burdens the CPU and increases its data exchange with memory. The memory‐wall issue and weak parallelism would become burdensome as the involved random number quantity is huge. For the above reasons, it seems extraordinarily valuable to develop hardware TRNG as well as the matching algorithm to transform the Bernoulli bits into any designed PDFs.

**Figure 3:**
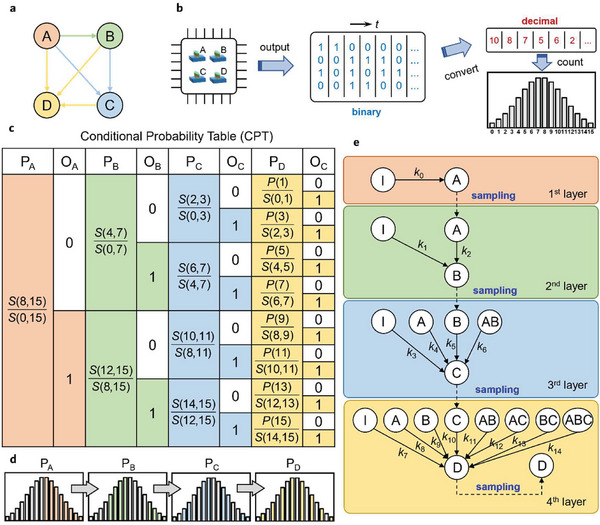
Schematic of the proposed PDF‐configurable true random number generators. a) The Bayesian network to generate a random number *N*. b) The process of generating random numbers with a desired PDF. c) the conditional probability table (CPT) used by the random number generator. Here *S*(*i*,*j*) = Σ*_i_^j^P*(*N*). d) The relationship between the conditional probabilities in c and the probabilities of the desired distribution. e) the network to generate true random numbers according to a designed PDF. It contains four layers, each of which samples one bit of random numbers A, B, C, and D as indicated by the dashed lines. The weights in the network can be deterministically preset according to the protocol in Section S2 (Supporting Information).

In order to generalize the Bernoulli bits to a continuous TRNG with a desired PDF, we consider a Bayesian network as shown in **Figure** **3**a. Here we schematically design a 4‐bit TRNG consisting of 4 SOT‐MTJs (Figure 3b). This TRNG can output 16 quasi‐continuous results in total, 0 or 1 … or 15. The resolution can be further improved by more bits. In the following, we design a protocol to make the TRNG produce 0–15 randomly according to a desired PDF, supposing the voltage‐dependence of *p* of the SOT‐MTJs is already known and the same for simplicity. The protocol can be interpreted by a 4‐node Bayesian network (Figure 3a) and its corresponding conditional probability table (CPT in Figure 3c). The nodes A, B, C, and D of the Bayesian network denote the first to fourth binary digit of a generated random number *N*.

(1) $$N=2^{3}A+2^{2}B+2^{1}C+2^{0}D$$

To produce a designed PDF, the 0 or 1 output probability *p*_B_ of the B node is decided by the output of the A node, *p*_B =_ *p*_B|A_. Thus, there should exist a directed edge from A to B. In the Bayesian network language, A (B) is the parent (child) node of B (A) as shown in Figure 3a. Correspondingly, the 0 or 1 output probability *p*_C_ of the C node is determined by the results of the A and B nodes, or *p*_C =_ *p*_C|A,B_. Thus, there exist two edges from A and B to C. The same argument is true for the edges directed to D. The conditional probability *p*_B|A_ and *p*_C|A,B_ and *p*_D|A,B,C_ are summarized in the CPT (Figure 3c).

Hereafter, we show the details of the generation process of random numbers. The desired PDF is discretely described as *P*(*N*) with a $\sum_{N\;=\;0}^{15}P\;\left(N\right)=\;1$. As shown by the CPT of the Bayesian network in Figure 3c, *p*_A_ for *A = *1 is $\sum_{N\;=\;8}^{15}P\left(N\right)$ by choosing suitable *V*_A_ according to the voltage‐dependence *f*_A_ of the switching probability of the A node, *V*_A =_ *f*_A_^−1^(*p*_A_). Here *f* ^−1^ denotes the corresponding inverse function of an *f* function. If the final output of the A node is 1, then *p*_B|A_ = $\sum_{N=12}^{15}P\left(N\right)\;/\sum_{N\;=\;8}^{15}P\left(N\right)$. Otherwise, as A = 0, *p*_B|A_ = $\sum_{N=4}^{7}P\left(N\right)\;/\sum_{N\;=\;0}^{7}P\left(N\right)$. At the hardware level, different *p*_B|A_ is implemented by selecting the proper *V*_B =_ *f*_B_^−1^(*p*_B|A_) of the B node according to the results of the A node. The selected voltages to drive the C and D nodes are also determined similarly, *V*_C =_ *f*_C_^−1^(*p*_C|AB_) and *V*_D =_ *f*_D_^−1^(*p*_D|ABC_). *p*_A_ and other conditional probabilities *p*_B|A_, *p*_C|AB_ and *p*_D|ABC_ can be retrieved from the CPT.

Worth noting, here the nonvolatility of the nodes matters due to the following two reasons: 1) the A, B and C nodes should keep their output of each until the outcome of the D node turns up in order to finally release a random number between 0 and 15; 2) more importantly, the result of the former node should be memorized and retrieved to cascade the switching event of the subsequent node since the switching condition of the latter is restricted by the conditional probability of the former as shown in Figure 3c–e. SOT‐MTJs with high barriers are exactly qualified in this nonvolatile sense.

When practically generating the 0–15 random numbers using the Bernoulli TRNGs, we may have to occasionally call some conditional probability values from the CPT which is remotely stored in some form. The invocation mode may be time‐consuming and energy‐inefficient. Here we provide an alternative, a network with four layers as shown in Figure 3e, to facilitate a local invocation of the CPT values. The corresponding weights can be explicitly obtained following the method in the Section S2 (Supporting Information). Note that this network protocol can also be easily implemented in a circuitry manner, which should be more efficient than the remote calling CPT method.

**Figure 4:**
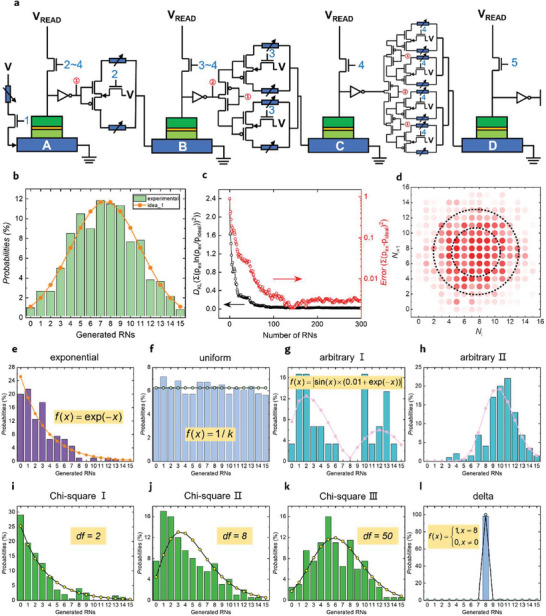
Results of the PDF‐configurable TRNGs based on SOT‐MTJs. a) A proposal for the circuitry implementation for cascading sampling of the Bernoulli bits A, B, C, and D. b) Realization of the Gaussian PDF. c) Divergence of the measured PDF with the desired PDF as a function of the generated random numbers. d) No correlation between generated neighboring random numbers *N_i_* and *N_i_*_+1_. e–h) The exponential PDF, uniform, two arbitrarily defined PDFs. i–k) The Chi‐square PDFs with various parameters *df* = 2, 8, and 50. l) the delta‐type PDF which means the TRNG can selectively output any numbers between 0 and 15 with almost 100% probability.

The above protocols can be implemented by four SOT‐MTJs and the corresponding variable resistors (**Figure** **4**a) which are used to store the resistance states of the former SOT‐MTJ nodes. The blue labels on the transistors represent the conduction sequence. In a continuous five‐step operation, the transistors are turned on in sequence, and remain off at all other times. For the second to fourth MTJs, the write voltage of each MTJ is influenced by the state of all previous MTJs. This circuit design implements the transfer of the conditional probabilities described in Figure 3c.

In our experiments, we demonstrate another way, using a single SOT‐MTJ by time‐division multiplexing, to construct the TRNG with four nodes. In this case, *f*_A =_ *f*_B =_ *f*_C =_ *f*_D_, loosening uniformity requirements for the SOT‐MTJs. First, if we set *P*(*N*) = 1/16 and the CPT becomes trivial, a uniform PDF is naturally obtained as shown in Figure 4f. For the BNNs, the Gaussian PDF is usually preferred. Here we also realized a Gaussian PDF as shown in Figure 4b. Figure 4c shows the Kullback–Leibler divergence *D*_KL = _Σ*p*_ex_·lg(*p*_ex_/*p*_ideal_) which is usually used to characterize the deviation of two PDFs, the experimentally sampled PDF *p*_ex_ and the ideal one *p*_ideal_. Another deviation defined as Σ(*p*_ex_‐*p*_ideal_)^2^ between the designed Gaussian PDF and the experimentally sampled PDF as a function of the output numbers is also shown. A fast reduction in both divergences is observed within 200 random numbers. In experiments, we thus generate 200 or above random numbers to evaluate the quality of various PDFs. The statistical independence of the generated neighboring random numbers *N_i_* and *N_i_*_+1_ is shown in Figure 4d. The horizontal and vertical coordinates are the adjacent random numbers, respectively. The redness indicates the probability of *N_i_*_+1_ following *N_i_* and the dashed circles are guided for eyes to show the circular symmetry of the data. For any *N_i_*, *N_i_*_+1_ seems symmetric regarding the axis of *N_i_*_+1 = _8, which indicates a weak correlation of *N_i_*_+1_ on *N_i_*.

Following the above protocol, we have also configurated the TRNG to other PDFs such as the exponential one (Figure 4e), Chi‐square PDFs with different parameters (Figure 4i–k), the delta one (Figure 4l) and even arbitrarily defined ones (Figure 4g,h), showing the flexibility, versatility, and programmability of these TRNGs. Here not only the variety of PDFs (Figure 4e–h) but also the same PDF with different controlling variables (the Chi‐square PDF with *df* = 2, 8 and 50 in Figure 4i,j) can be reconfigured using the same SOT‐MTJs, which improves the extensibility in applications of the TRNGs. Note 1) the above PDFs or other arbitrarily defined PDFs can play roles in various kinds of simulations according to all sorts of applications, which is worthy of exploring by communities in the future. For example, the Gaussian PDFs are widely applied in BNNs, Monte Carlo algorithms for communications channel modeling and financial modeling, etc.^[^ ^12b,d,e^ ^]^ The Chi‐square PDFs with different parameters are applied in testing the machines of all kinds of curve fittings and the independence of two variables. 2) A same SOT‐MTJ can be configured to give out various PDFs, showing the powerfulness of this kind of TRNGs based on the high‐barrier SOT‐MTJs.

Note that some classic algorithms can also help to realize any desired PDFs *p*(*x*). One typical algorithm uses the reverse function *x = *CDF^−1^(*y*) of cumulative distribution functions (CDF) that are integral of the PDFs. One first uniformly samples *y* within [0, 1], then computes the reverse function *x = *CDF^−1^(*y*) and finally obtains the designed occurrence of *x* according to the designed *p*(*x*). This approach not only requires a computable CDF^−1^, which may be difficult in some cases but also needs the involvement of many CPU operations, addition and comparison between floating numbers, as well as data transfer between CPU and the random number generators. For the above two reasons, the conventional CDF method is relatively inefficient. Nevertheless, the method proposed here is to use the simplest Bernoulli random numbers, binary random numbers, to convert it directly into PDF configurable random numbers, which is closer to the underlying binary logics of the hardware and benefits to improve speed and efficiency.

Another example is the acceptance–rejection sampling: sampling *x* accords to a proposal PDF *q*(*x*) and accepts or rejects the sampling result according to an acceptance probability *α*(*x*)≡*p*(*x*)/[*kq*(*x*)]; then one obtains the designed occurrence of *x* ideally coinciding with the designed PDF *p*(*x*). Here *k*, as a scaling constant, assures *p*(*x*)≤*kq*(*x*) for all *x*. The acceptance–rejection sampling accepts samples only with a certain probability. In the case of high‐dimensional and complex distributions, the efficiency of the acceptance–rejection sampling can be low. Besides, it is not easy to find an appropriate *k* and *q*(*x*). In contrast, every sampling result in our work is not wasted and is meaningful, improving its sampling efficiency. More importantly, while the above two algorithms sample a uniform or a pre‐proposed PDF *q*(*x*), here our used algorithm directly samples the desired *p*(*x*) without additional computational burden, which can ease its hardware implementation.

## 3 Conclusion

In summary, high barrier SOT‐MTJs with 300 ps writing speed, over 10^12^ endurance, above 100% TMR ratio and 50 × 200 nm sizes were developed and further used as switching‐probability‐tunable true random number generators, making them an excellent Bernoulli bit at the hardware level. Furthermore, to accord with the binary and tunable features of the Bernoulli bit, we develop a method based on the Bayesian network to give out arbitrary probability‐distribution functions in any resolutions. In the experiment, we applied the time‐division multiplexing method to realize the 4‐bit random number generators whose PDFs can be configurated as desired into the Gaussian, delta, uniform, Chi‐square, exponential, and other custom‐defined PDFs. These PDF‐definable TRNGs can be widely used in many hardware accelerators such as the Mento Carlo sampling and simulations, simulated annealing and Bayesian neuron networks, which also helps to broaden the application scopes of the SOT‐MTJ devices.

## 4 Experimental Section

The used SOT‐MTJ stack was W(3)/CoFeB(1.4)/MgO(1.5)/CoFeB(3)/W(0.4)/Co(2.8)/ IrMn(10)/Ru(4) with the nominal thickness values in parentheses in nanometers. The film was grown on thermally oxidized silicon substrates by magnetron sputtering in a vacuum environment of 10^−6^ Pa. Subsequently, annealing was performed in an in‐plane magnetic field of 0.8 T at 380 °C for 30 min. The film was then patterned into SOT‐MTJ devices using the standard electron‐beam lithography and dry etching process as in ref. [16]. The magneto‐electro transport measurements were conducted at room temperature with the devices placed in a Helmholtz coil and connected to the testing instruments using four tungsten steel probes. Worth mentioning, here the MTJ had in‐plane uniaxial magnetic anisotropy; the spin current via the spin Hall effect in the W channel was polarized collinear to the in‐plane easy axis, which made its SOT switching inherently field‐free.

## Conflict of Interest

The authors declare no conflict of interest.

## Supporting information

Supporting Information

## Data Availability

The data that support the findings of this study are available from the corresponding author upon reasonable request.

## References

[1] S. Misra , L. C. Bland , S. G. Cardwell , J. A. C. Incorvia , C. D. James , A. D. Kent , C. D. Schuman , J. D. Smith , J. B. Aimone , Adv. Mater. 2022, 35, 2204569. 10.1002/adma.202204569 36395387

[2] T. Dalgaty , N. Castellani , C. Turck , K.‐E. Harabi , D. Querlioz , E. Vianello , Nat. Electron. 2021, 4, 151.

[3] a) W. Beker , A. Wołos , S. Szymkuć , B. A. Grzybowski , Nat. Mach. Intell. 2020, 2, 457;

[4] T. Albash , D. A. Lidar , Phys. Rev. X 2018, 8, 031016.

[5] a) Z. Ghahramani , Nature 2015, 521, 452; 26017444 10.1038/nature14541

[6] A. Merolla Paul , V. Arthur John , R. Alvarez‐Icaza , S. Cassidy Andrew , J. Sawada , F. Akopyan , L. Jackson Bryan , N. Imam , C. Guo , Y. Nakamura , B. Brezzo , I. Vo , K. Esser Steven , R. Appuswamy , B. Taba , A. Amir , D. Flickner Myron , P. Risk William , R. Manohar , S. Modha Dharmendra , Science 2014, 345, 668. 25104385 10.1126/science.1254642

[7] a) S. Chib , E. Greenberg , American Statist. 1995, 49, 327;

[8] F. James , Comput. Phys. Commun. 1990, 60, 329.

[9] a) B. Sunar , W. J. Martin , D. R. Stinson , IEEE Trans. Comp. 2007, 56, 109;

[10] a) T. Jennewein , U. Achleitner , G. Weihs , H. Weinfurter , A. Zeilinger , Rev. Sci. Instrum. 2000, 71, 1675; 10.1103/PhysRevLett.84.4729 10990782

[11] a) L. Yin , R. Cheng , Y. Wen , C. Liu , J. He , Adv. Mater. 2021, 33, 2007081; 10.1002/adma.202007081 34105195

[12] a) H. H. Thodberg , IEEE Trans. Neural Netw. 1996, 7, 56; 18255558 10.1109/72.478392

[13] a) P. Li , A. Chen , D. Li , Y. Zhao , S. Zhang , L. Yang , Y. Liu , M. Zhu , H. Zhang , X. Han , Adv. Mater. 2014, 26, 4320; 24752966 10.1002/adma.201400617

[14] B. Zhang , Y. Liu , T. Gao , D. Zhang , W. Zhao , L. Zeng , presented at 2021 IEEE International Electron Devices Meeting (IEDM), IEEE, San Francisco, CA, USA, 2021.

[15] G. E. Box , M. E. Muller , Annals Mathematical Statistics 1958, 29, 610.

[16] M. K. Zhao , R. Zhang , C. H. Wan , X. M. Luo , Y. Zhang , W. Q. He , Y. Z. Wang , W. L. Yang , G. Q. Yu , X. F. Han , Appl. Phys. Lett. 2022, 120, 182405.

