# Supplementary material for: Probability‐Distribution‐Configurable True Random Number Generators Based on Spin‐Orbit Torque Magnetic Tunnel Junctions
Source: Adv Sci (Weinh). 2024 Apr 15;11(23):2402182. doi: 10.1002/advs.202402182 (PMC11186041; doi:10.1002/advs.202402182)
Supplement: Supplementary file 1 — Supporting Information [file ADVS-11-2402182-s001.pdf]

ADVANCED SCIENCE
Open Access

Supporting Information

for *Adv. Sci.*, DOI 10.1002/advs.202402182

Probability-Distribution-Configurable True Random Number Generators Based on Spin-Orbit Torque Magnetic Tunnel Junctions

Ran Zhang, Xiaohan Li, Mingkun Zhao, Caihua Wan, Xuming Luo, Shiqiang Liu, Yu Zhang, Yizhan Wang, Guoqiang Yu and Xiufeng Han**

Supporting Information

Probability-Distribution-Configurable True Random Number Generators Based on Spin-Orbit Torque Magnetic Tunnel Junctions

Ran Zhang, Xiaohan Li, Mingkun Zhao, Caihua Wan, Xuming Luo, Shiqiang Liu, Yu Zhang, Yizhan Wang, Guoqiang Yu and Xiufeng Han**

Supporting Information 1. Randomness Test

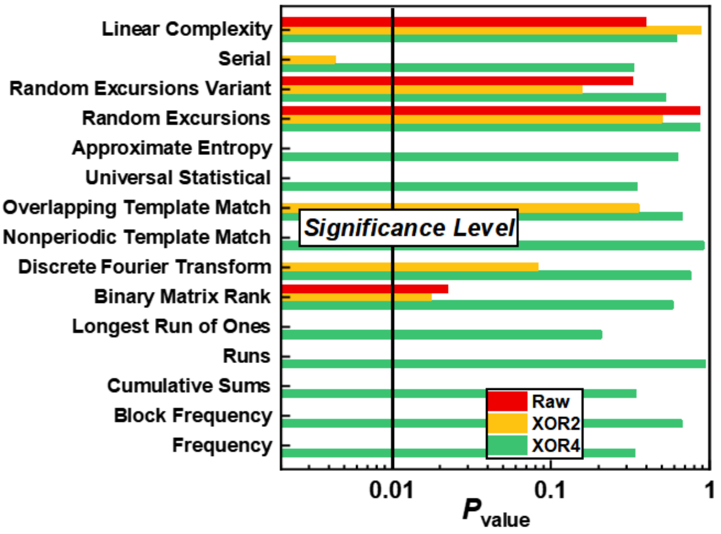

Figure S1. Results of the NIST 800-22 Randomness Test Suit for the generated random numbers by the TRNG with switching probability being 50%.

Supporting Information 2. Determination method of the network weights in Figure 3d

The information in the conditional probability table can be remotely retrieved by the true random number generators via dispatching of a CPU. However, the CPT can also be translated as a sequential running of a network with its edge weights predetermined by the CPT as shown in Figure 3**d**. In this case, all the needed information to give out the desired PDF can be retrieved locally without remote

invocation, which should in principle improve efficiency. The weights in the Figure should be preset as following.

For the first layer,

$$P(A) = k_0 = \frac{\sum_{i=8}^{15} P(i)}{\sum_{i=0}^{15} P(i)} = \sum_{i=8}^{15} P(i).$$

For the second layer,

$$k_1 + k_2 A = P(B|A)$$

As A=1 or 0, *P*(B|A) has different forms. Thus, we can obtain the following values of k_1 and k_2.

$$k_1 + k_2 = P(B|A=1) = \frac{\sum_{i=12}^{15} P(i)}{\sum_{i=8}^{15} P(i)}$$

$$k_1 = P(B|A=0) = \frac{\sum_{i=4}^{7} P(i)}{\sum_{i=0}^{7} P(i)}$$

$$k_2 = \frac{\sum_{i=12}^{15} P(i)}{\sum_{i=8}^{15} P(i)} - \frac{\sum_{i=4}^{7} P(i)}{\sum_{i=0}^{7} P(i)}$$

For the third layer,

$$k_3 I + k_4 A + k_5 B + k_6 AB = P(C|AB).$$

The above expression has not only considered the influence of A or B independently on C but also their cooperative impact on the probability of C. The value of $k_{3,4,5,6}$ can be determined as following.

$$\begin{bmatrix} k_3 \\ k_4 \\ k_5 \\ k_6 \end{bmatrix} = \begin{bmatrix} 1 & 1 & 1 & 1 \\ 1 & 1 & 0 & 0 \\ 1 & 0 & 1 & 0 \\ 1 & 0 & 0 & 0 \end{bmatrix}^{-1} \begin{bmatrix} P(C|A=1,B=1) \\ P(C|A=1,B=0) \\ P(C|A=0,B=1) \\ P(C|A=0,B=0) \end{bmatrix}$$

Here,

$$P\left(C\middle|A=1,B=1\right)=\frac{P(14)+P(15)}{P(12)+P(13)+P(14)+P(15)}$$

$$P\left(C\middle|A=1,B=0\right)=\frac{P(10)+P(11)}{P(8)+P(9)+P(10)+P(11)}$$

$$P\left(C\middle|A=0,B=1\right)=\frac{P(6)+P(7)}{P(4)+P(5)+P(6)+P(7)}$$

$$P\left(C\middle|A=0,B=0\right)=\frac{P(2)+P(3)}{P(0)+P(1)+P(2)+P(3)}$$

They can be obtained from the CPT accordingly.

For the fourth layer, similarly, considering the mutual and multivariable interactions,

$$k_7 I + k_8 A + k_9 B + k_{10} C + k_{11} AB + k_{12} AC + k_{13} BC + k_{14} ABC = P\left(D\middle|ABC\right).$$

$$\begin{bmatrix} k_7 \\ k_8 \\ k_9 \\ k_{10} \\ k_{11} \\ k_{12} \\ k_{13} \\ k_{14} \end{bmatrix} = \begin{bmatrix} 1 & 1 & 1 & 1 & 1 & 1 & 1 & 1 \\ 1 & 1 & 1 & 0 & 1 & 0 & 0 & 0 \\ 1 & 1 & 0 & 1 & 0 & 1 & 0 & 0 \\ 1 & 1 & 0 & 0 & 0 & 0 & 0 & 0 \\ 1 & 0 & 1 & 1 & 0 & 0 & 1 & 0 \\ 1 & 0 & 1 & 0 & 0 & 0 & 0 & 0 \\ 1 & 0 & 0 & 1 & 0 & 0 & 0 & 0 \\ 1 & 0 & 0 & 0 & 0 & 0 & 0 & 0 \end{bmatrix}^{-1} \begin{bmatrix} P\left(D\middle|A=1,B=1,C=1\right) \\ P\left(D\middle|A=1,B=1,C=0\right) \\ P\left(D\middle|A=1,B=0,C=1\right) \\ P\left(D\middle|A=1,B=0,C=0\right) \\ P\left(D\middle|A=0,B=1,C=1\right) \\ P\left(D\middle|A=0,B=1,C=0\right) \\ P\left(D\middle|A=0,B=0,C=1\right) \\ P\left(D\middle|A=0,B=0,C=0\right) \end{bmatrix}$$

Here, also according to the CPT,

$$P\left(D\middle|A=1,B=1,C=1\right)=\frac{P(15)}{P(14)+P(15)}$$

$$P\left(D\middle|A=1,B=1,C=0\right)=\frac{P(13)}{P(12)+P(13)}$$

$$P\left(D\middle|A=1,B=0,C=1\right)=\frac{P(11)}{P(10)+P(11)}$$

$$P\left(D\middle|A=1,B=0,C=0\right)=\frac{P(9)}{P(8)+P(9)}$$

$$P\left(D\middle|A=0,B=1,C=1\right)=\frac{P(7)}{P(6)+P(7)}$$

$$P\left(D\middle|A=0,B=1,C=0\right)=\frac{P(5)}{P(4)+P(5)}$$

$$P\left(D\middle|A=0,B=0,C=1\right)=\frac{P(3)}{P(2)+P(3)}$$

$$P\left(D\middle|A=0,B=0,C=0\right)=\frac{P(1)}{P(0)+P(1)}\quad .$$

Using the values of $P(N)$ from a desired PDF, we can obtain the network weights following the above protocol. And further following the network in Figure 3**d**, we can generate the random numbers in between 0 and 15 which satisfy the designed PDF. The above protocol is better than the remote CPT because we can use the former generated random numbers to locally cascade the generation of the following random numbers without remote calling the CPT values. Furthermore, the network can be in principle implemented in some hardware or circuitry form, which is more efficient.
